# High performance antenna-on-chip inspired by SIW and metasurface technologies for THz band operation

**DOI:** 10.1038/s41598-022-27364-8

**Published:** 2023-01-02

**Authors:** Mohammad Alibakhshikenari, Bal S. Virdee, Renu Karthick Rajaguru, Amjad Iqbal, Muath Al‑Hasan, Chan H. See, Francisco Falcone

**Affiliations:** 1 Department of the Signal Theory and Communications, Universidad Carlos III de Madrid, 28911 Leganés, Madrid Spain; 2 Center for Communications Technology, London Metropolitan University, London, UK; 3 Institut National de La Recherche Scientifique (INRS), Montréal, QC H5A1K6 Canada; 4 Department of Network and Communications Engineering, Al Ain University, 64141, Al Ain, UAE; 5 School of Engineering & the Built Environment, Edinburgh Napier University, 10 Colinton Rd., Edinburgh, EH10 5DT UK; 6 Department of Electric, Electronic and Communication Engineering and the Institute of Smart Cities, Public University of Navarre, 31006 Pamplona, Spain; 7 School of Engineering and Sciences, Tecnologico de Monterrey, 64849 Monterrey, Mexico

**Keywords:** Engineering, Electrical and electronic engineering

## Abstract

In this paper, a high-performance antenna-on-chip (AoC) is implemented on gallium arsenide (GaAs) wafer based on the substrate integrated waveguide (SIW) and metasurface (MTS) technologies for terahertz band applications. The proposed antenna is constructed using five stacked layers comprising metal-GaAs-metal-GaAs-metal. The conductive electromagnetic radiators are implemented on the upper side of the top GaAs layer, which has a metallic ground-plane at its underside. The metallic feedline is implemented at the underside of the bottom GaAs layer. Dual wrench-shaped radiators are framed by metallic vias connected to the ground-plane to create SIW cavity. This technique mitigates the surface waves and the substrate losses, thereby improving the antenna’s radiation characteristics. The antenna is excited by a T-shaped feedline implemented on the underside of the bottom GaAs substrate layer. Electromagnetic (EM) energy from the feedline is coupled to the radiating elements through the circular and linear slots etched in the middle ground-plane layer. To mitigate the surface-wave interactions and the substrate losses in the bottom GaAs layer, the feedline is contained inside a SIW cavity. To enhance the antenna’s performance, the radiators are transformed into a metamaterial-inspired surface (i.e., metasurface), by engraving periodic arrangement of circular slots of sub-wavelength diameter and periodicity. Essentially, the slots act as resonant scatterers, which control the EM response of the surface. The antenna of dimensions of 400 × 400 × 8 μm^3^ is demonstrated to operate over a wide frequency range from 0.445 to 0.470 THz having a bandwidth of 25 GHz with an average return-loss of − 27 dB. The measured average gain and radiation efficiency are 4.6 dBi and 74%, respectively. These results make the proposed antenna suitable for AoC terahertz applications.

## Introduction

Considerable attention has been directed towards terahertz (THz) imaging systems, because this frequency band (0.1–10 THz) has unique spectroscopic properties for discriminating various materials. THz signal that can leverage advantages of both millimeter-wave and optics, such as high spatial resolution, good penetration depth to dielectric material or human tissue with no harmful ionization^1^. The exponential growth in data traffic in wireless communication systems has necessitated research at THz band as it can support significantly higher data rates of several Tbps than millimeter-wave band^2,3^. Maintaining the status quo of existing wireless communications infrastructure is otherwise likely to cripple it. Compared with optical communication, the THz communication system is insensitive to the atmospheric effects in outdoor communications and it’s relatively easily to track its beam. Moreover, THz systems can enhance the link gain by exploiting reflective paths.

On-chip antennas are needed to mitigate against the large transitional losses experienced by using interconnects at terahertz frequencies^4^. Antenna size for on-chip applications must meet more stringent requirements than their off-chip counterparts. At frequencies greater than 200 GHz, the size of the λ/4 or λ/2 antenna is small enough to fit onto a chip. The two types of antennas, which are commonly used for on-chip designs are patch antennas^5–7^, and dipole antennas^8,9^. These structures have sharp but narrow bandwidth that can limit the data throughput of a chip^10^. Typically, a patch antenna, when excited using a single-ended feedline, radiates energy in its broadside. The gain of such antennas for on-chip applications is limited to about 1 dB and the radiation efficiency to ~ 25% because a metallic ground layer is used to shield the radiator and lossy silicon substrate. The antenna’s fractional bandwidth is constrained to ~ 2.5%. This is mainly due to the gap between on-chip radiator and the ground layer. On the other hand, dipole antennas radiate energy omnidirectionally, and can be excited differentially. Although on-chip dipole antennas exhibit advantages of compact size and broad bandwidth (~ 15%), however they exhibit a relatively low gain (~ 8 dBi), and poor radiation efficiency (~ 10%). This is because a great proportion of the radiated power is dissipated in the silicon substrate. The shortcomings of dipole antennas can be overcome by placing a dielectric focal lens over the antenna^11,12^. On-chip antennas based on dielectric focal lens have a typical gain and radiation efficiency of ~ 15 dBi and ~ 60%, respectively. However, this technique introduces additional cost for system integration involving alignment of the antenna at the focus point of the focal lens. Furthermore, backside excitation is necessary so that the antenna can be attached on the surface of the focal lens using high-resistivity silicon substrate to minimize the substrate absorption.

Substrate integrated waveguide (SIW) technology has been used to design high-Q components in both millimeter and sub-THz bands^13–17^. Such structures combine the benefits of both planar transmission-line and non-planar waveguide with lower losses and wideband performance. SIW is essentially a dielectric cavity with metal layers on the top and bottom surfaces, surrounded by metallic walls. SIW antennas have been proposed at chip scale^17^; however, they have low gain and suffer from narrow bandwidths. This is evident in^17^, where a 400 GHz on-chip SIW antenna fabricated in SiGe process has a gain of 0.55 dBi, and a relative bandwidth of 7.8%.

Metasurface (MTS) is a two-dimensional (2D) metamaterial with subwavelength thickness^18,19^. MTS engineer EM-waves impinging on its surface to undergo abrupt amplitude, phase, and polarization changes. A metasurface can be constructed from a periodic array of well-designed metallic scattering geometries of sub-wavelength dimensions that are created on the surface of an electromagnetically transparent substrate. Low profile microstrip patch radiators based on MTS have been shown to provide a wide bandwidth and enhanced gain performance^20–28^. In many antenna configurations, the metasurface is placed directly above^20,21^ or under^22,23^ the radiator, with an air-gap separation^24^. The drawback of these antennas includes complex design, large profile, and poor mechanical properties. It has been shown in^25–28^ that by stacking MTS on the radiator without an air gap a low-profile antenna can be realized with a broad bandwidth (|S_11_|≤ –10 dB) and a high gain. At THz frequency, the small physical size of the unit cell scatters precludes the design of complex structures from a fabrication point of view^29^.

This paper demonstrates the advantage of integrating SIW and MTS technologies in the design of on-chip antennas implemented on Gallium Arsenide (GaAs) substrate. The proposed antenna structure is far less complex to fabricate using the existing technology and should enable a significant cost reduction. In addition, the use of SIW technology makes it possible to integrate all transceiver components, thereby alleviating transitional losses experienced by using interconnects at THz frequencies.

This paper is organized as follows. Discussed in “next” Section is SIW inspired THz antenna-on-chip. In Section “AOC based on amalgamating SIW and MTS technologies”, SIW and MTS technologies are integrated in the design of AoC. In Section “Impedance matched feedline of the SIW-MTS inspired AOC”, the feedline is transformed to a metasurface to enhance the performance of the AoC. In Section “State-or-the-art comparison”, the salient features of the antenna are compared to state-of-the-art THz antennas. The work is concluded in “Conclusion” Section.

## SIW-inspired THz antenna-on-chip

This section introduces the first version of the proposed antenna-on-chip design, implemented on GaAs, and involving SIW-based cavities employed to mitigate the coupling effects induced by the surface waves.

### Antenna geometry and operating principles

The proposed antenna-on-chip is constructed on a stack of five layers that comprise metal-substrate-metal-substrate-metal. The radiation patches are implemented on the top surface of the upper Gallium Arsenide substrate layer. The bottom surface of the upper GaAs layer is metallized and used as a common ground plane. Immediately below, there is a lower second GaAs layer. The feedline is implemented on the bottom surface of the lower GaAs layer. The GaAs used had a relatively high permittivity (*ε*_*r*_) of 12.88, which was necessary to reduce the overall antenna size. The loss tangent of GaAs substrate is 0.0004. Authors in^30^ have measured the dielectric properties of GaAs across 0.2–1.5 THz using time domain spectroscopy. Their results show that over this frequency range the change in the relative permittivity is negligible however the loss tangent increases by a factor of nine. A bulk GaAs technology process is used here to reduce the manufacturing costs.

**Figure 1:**
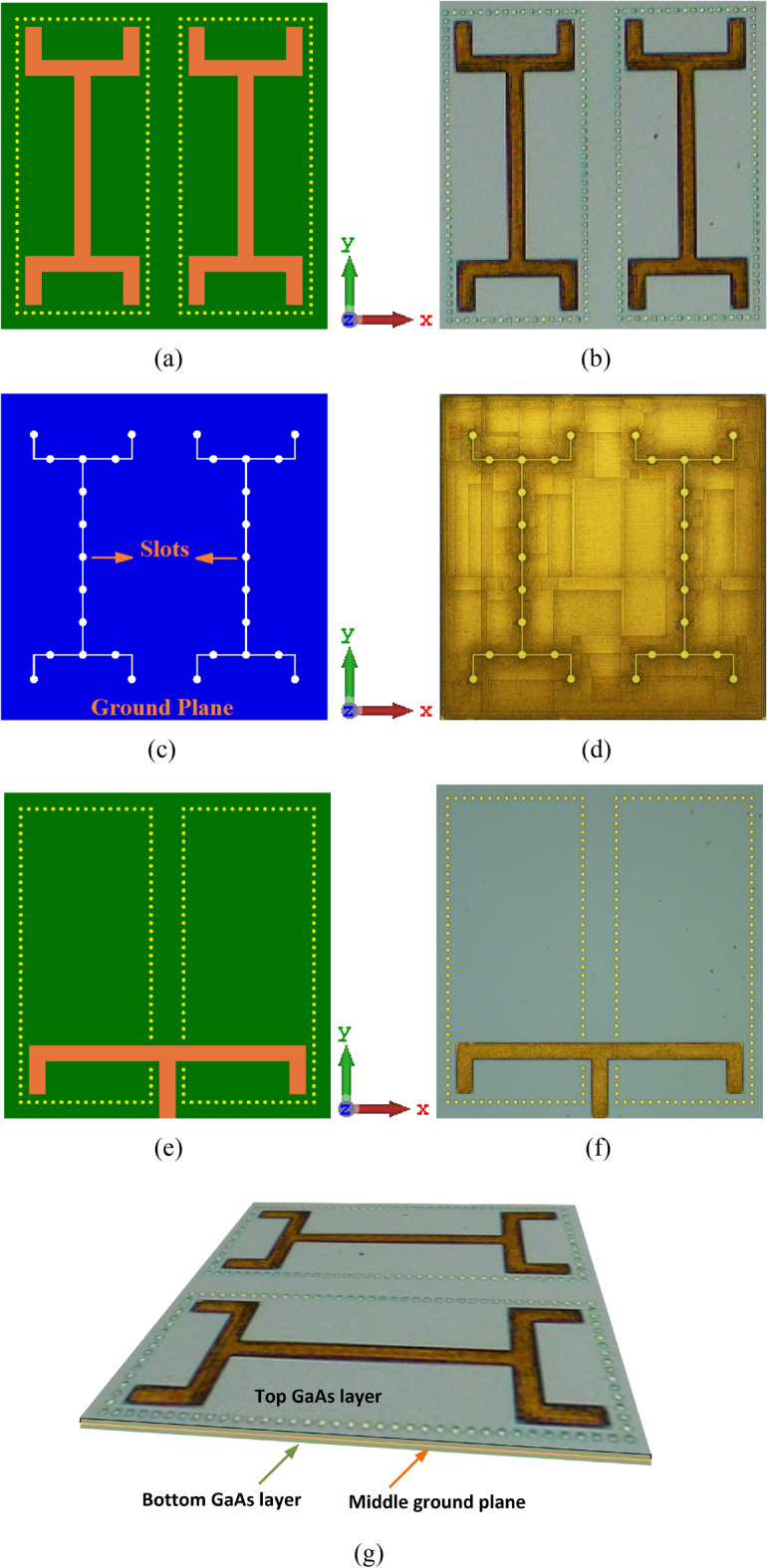
Layout of the SIW inspired on-chip antenna, (**a**) simulated configuration and (**b**) fabricated prototype of the GaAs antenna showing the top surface of the top GaAs layer; (**c**) simulated layout and (**d**) fabricated prototype of the middle metallic ground-plane with circular and linear slots. This layer is sandwiched between the top (**b**) and bottom (**f**) GaAs layers; (**e**) simulated configuration; (**f**) fabricated prototype of the GaAs antenna showing the bottom surface of the bottom GaAs layer; and (**g**) isometric view of the on-chip antenna.

The two wrench-shaped radiation elements created on the top side of the upper GaAs layer is shown in Fig. 1a,b. The configuration of the wrench-shaped antenna was selected as it provides excellent radiation coverage in both orthogonal planes over an ultra-wideband frequency range^31^. Surface wave modes are excited in the antenna structure that contribute to losses.

Moreover, the surface wave interactions between the two radiators can adversely affect the radiation characteristics of the on-chip antenna^32^. To mitigate the effects of surface waves, each radiator is confined using the SIW technology, which is realized by punching a periodic array of lateral via-holes through the upper GaAs layer to the middle ground-plane. The via-holes are filled with metal posts^33^.

The feed mechanism to excite the AoC is from the underside of the antenna structure using a T-shaped structure, as shown in Fig. 1e,f. The feed structure is constructed on the underside of the bottom GaAs layer. T-shaped feedline was used as it provides broadband matching^34^. SIW was applied to the feedline structure to confine the electromagnetic field. The antenna is excited by coupling the EM energy from the bottom GaAs layer to the radiators on the top surface of the upper GaAs layer through the arrangement of slots, shown in Fig. 1c,d, implemented in the middle ground-plane layer. Arrangement of the circular and linear interconnected slots conform to the shape of wrench to enable a strong and optimum path for EM coupling via the ground-plane.

The feeding structure has been optimized for impedance matching between the input to the antenna using CST Microwave Studio, which is based on finite integration technique (FIT). The top and bottom of the fabricated prototype antenna are shown in Fig. 1b,f, respectively. The structural parameters of the antenna are listed in the Table 1 in terms of microns and guide-wavelength centered at 0.455 THz. The antenna dimensions are 400 × 400 × 8 μm^3^.

**Table 1:** Structural parameters of the antenna (in microns & guide-wavelength).

Radius of framing wall via-holes	2.5 μm	0.0136 *λ*_*g*_
Radius of feedline vias-holes	5 μm	0.0272 *λ*_*g*_
Radius of ground-plane slots	5 μm	0.0272 *λ*_*g*_
Width of the ground-plane slot lines	2 μm	0.0109 *λ*_*g*_
Gap between the framing wall vias-holes	2.5 μm	0.0136 *λ*_*g*_
Length of the radiation elements	340 μm	1.8507 *λ*_*g*_
Width of the radiation elements	20 μm	0.1088 *λ*_*g*_
Length of the feedline	340 μm	1.8507 *λ*_*g*_
Width of the feedline	20 μm	0.1088 *λ*_*g*_
Thickness of the GaAs substrate	3.5 μm	0.0191 *λ*_*g*_
Patches, GND plane, feeding network thickness	0.33 μm	1.7962 *λ*_*g*_
AoC dimensions	400 × 400 × 8 (μm)^3^	2.18 × 2.18 × 0.04 (*λ*_*g*_)^3^

### Simulation results and experimental validation

**Figure 2:**
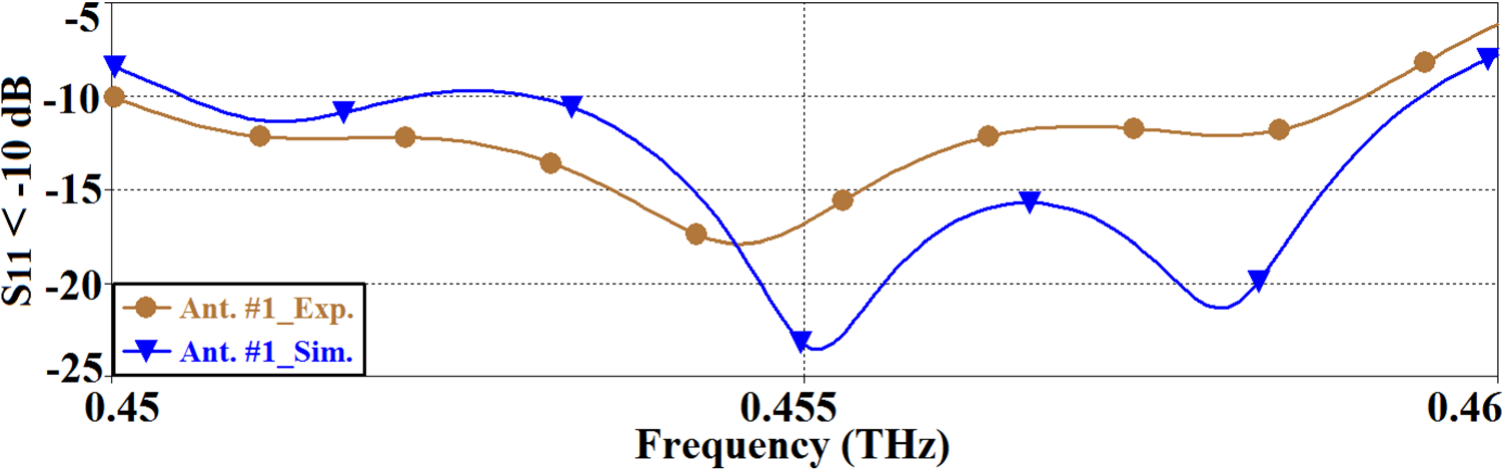
Simulated and measured reflection coefficient response of the SIW inspired antenna (Ant. #1).

The reflection-coefficient of the SIW inspired antenna is shown in Fig. 2. The simulated and measured impedance bandwidth of the antenna for |*S*_11_|≤ − 10 dB span from 0.451 to 0.459 THz, and from 0.450 to 0.458 THz, respectively. In both cases, the bandwidth is 8 GHz, which shows that there is good coherency between the simulated and the measured results. The average magnitude of the impedance match for the simulated and measured results are − 17.5 dB and − 14 dB, respectively. The measured reflection coefficient is better than the simulation across 0.45–0.454 THz however the measured results are worse than the simulation across 0.454–0.46 THz. The discrepancy is attributed to the imprecise simulation modelling.

**Figure 3:**
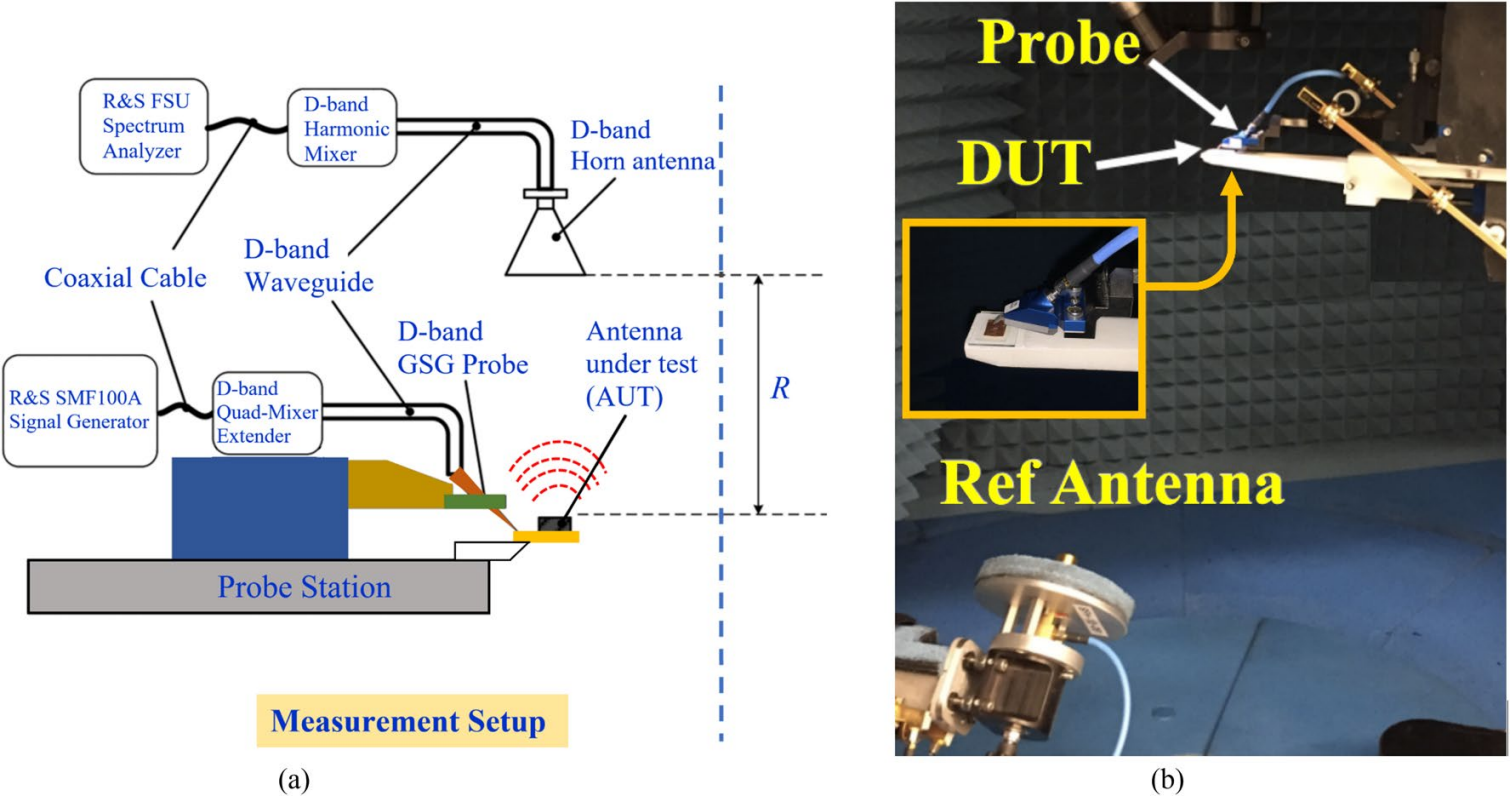
(**a**) Diagram of the antenna-on-chip (AoC) wafer measurement setup, and (**b**) Photograph of the AoC gain measurement setup.

**Figure 4:**
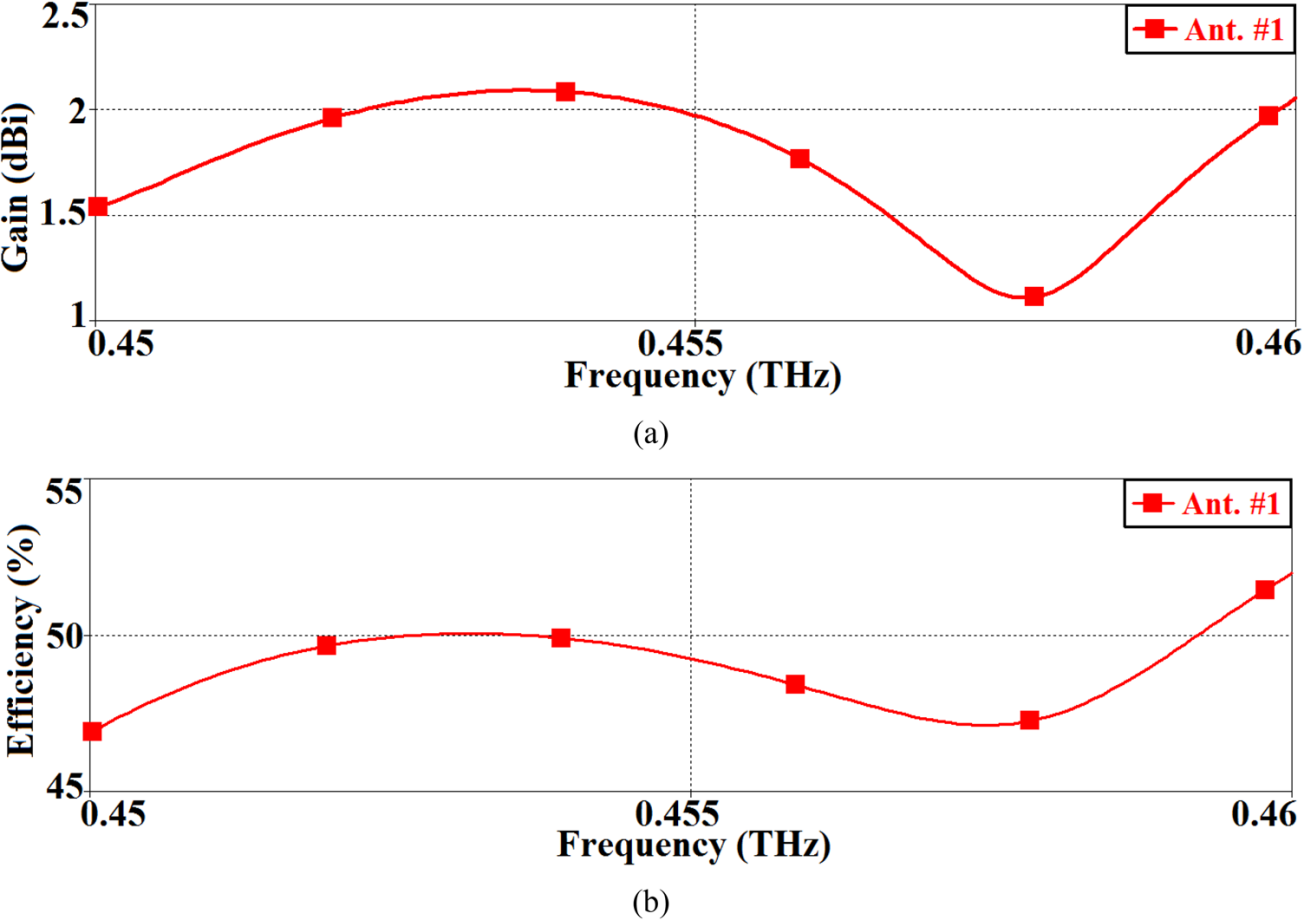
The measured radiation properties of the of the SIW inspired AoC (Ant. #1), (**a**) gain, and (**b**) radiation efficiency.

The antenna gain and radiation efficiency were measured using the setup shown in Fig. 3a. The photograph of the measurement setup is shown in Fig. 3b. Signal source from the Rohde & Schwarz SMF 100A was injected into a D-band quad-mixer extender. The transmission signal from the mixer was applied to the AoC with a D-band waveguide-to-GSG probe. The received signal at the D-band standard horn antenna was down-converted through a harmonic mixer and fed to the Rohde & Schwarz SMF Spectrum Analyzer. The horn antenna was used to measure the radiation from the AoC. The AoC was then replaced by another standard horn antenna, and the antenna gain was determined using the traditional method of comparing the power received by the standard horn of a known gain with that received by the AoC. The distance between the AoC and the reference antenna had to satisfy the far-field condition, which is equal to or greater than $r \ge 2D^{2} /\lambda_{o}$, where D is the largest aperture dimensions of the antenna and *λ*_*o*_ is the free-space wavelength at the operating frequency. The radiation efficiency of the AoC was calculated by taking the ratio of the measured radiated power to the input power. Measured antenna gain and the radiation efficiency over its operational frequency range are shown in Fig. 4. The gain and efficiency fluctuate between 1.1 and 2.1 dBi, and 47% and 52.5%, respectively. The average gain and efficiency are 1.6 dBi and 48%, respectively.

The above results corroborate that despite its small size the proposed SIW-inspired GaAs antenna operates over a wide frequency range at the terahertz band. In the next section, the metasurface technology is applied to enhance the antenna characteristics while maintaining its dimensions.

## AoC based on amalgamating SIW and MTS technologies

This section discusses an enhanced version of the antenna introduced in “SIW-inspired THz antenna-on-chip” Section, which is realized with metasurface technology (MTS).

### Antenna geometry and theoretical analysis

**Figure 5:**
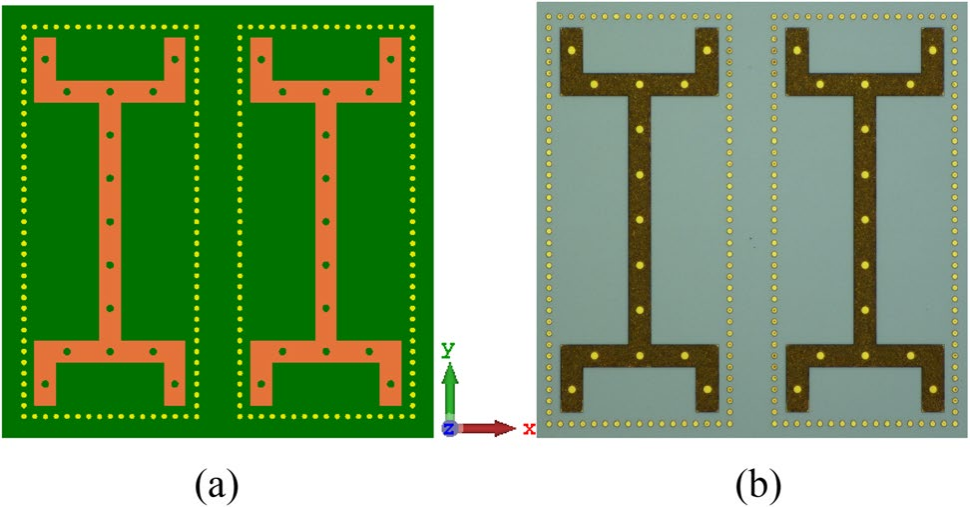
Configuration of the proposed SIW-MTS inspired AoC, (**a**) layout, and (**b**) fabricated GaAs prototype.

In this section, we have applied metasurface technology to enhance the performance of the SIW antenna presented in “SIW-inspired THz Antenna-on-Chip” Section. MTS was realized by etching periodic array of circular slots on the wrench-shaped radiation element, as shown in Fig. 5. The periodicity of the slots and the diameter of the slots are sub-wavelength at the operating frequency range of the THz antenna.

The distribution of individual scatterers is pivotal in determining the response of a surface. This property differentiates the metasurfaces from frequency selective surfaces, where periodicity of the scatters is of the order of the operating wavelength. The circular slots are aligned with the circular slots created on the ground-plane layer to maximize coupling of EM energy from the feedline on the underside of the bottom layer.

The circular slots etched on the radiating elements are non-magnetic scatterers resting on the GaAs substrate. The scatterers’ thickness is much smaller than the wavelength of the THz signal of interest. The metasurface locally modifies the amplitude, phase, or polarization of the incident light transmission or reflection.

For weakly-coupled scatterers, which are employed here, the metasurface can be modelled as a surface with spatially dependent local reflection and transmission coefficients. At the lossy metasurface, the sum of the transmitted and reflected powers is equal to, or smaller than the incident power, that is^35^

1 $$ \left| {t_{\parallel } } \right|^{2} + \left| {r_{\parallel }^{2} } \right| + \left| {t_{ \bot } } \right|^{2} + \left| {r_{ \bot } } \right|^{2} \le 1 $$

2 $$r_{\parallel } = \sqrt {\frac{{n_{1} }}{{n_{1} }}}t_{\parallel } - 1$$

3 $$ r_{ \bot } = \sqrt {\frac{{n_{1} }}{{n_{2} }}} t_{ \bot } $$

where the transmission and reflection coefficients in orthogonal polarization to the signal are represented by *t*_⊥_ and *r*_⊥_, whereas in the same polarization as the signal, by *t*_∥_ and *r*_∥_. The refractive indices of the GaAs substrate and air are *n*_2_ and *n*_1_, respectively. When the signal is incident at an angle *θ*_*i*_ with respect to the interface’s normal direction to the metasurface, Eq. (1) is still valid, while Eq. (2) and (3) are modified as

4 $$ r_{\parallel } = \sqrt {\frac{{n_{1} cos{ }\theta_{i} }}{{n_{2} cos{ }\theta_{r} }}} t_{\parallel } - 1 $$

5 $$ r_{ \bot } = \sqrt {\frac{{n_{1} cos{ }\theta_{i} }}{{n_{2} cos{ }\theta_{r} }}} t_{ \bot } $$

For a lossy metasurface, the left-hand-side of (1) is equal to 1 − *L*, where *L* is the fraction of the signal absorbed by the metasurface. This indicates that the loss resulting from the material absorption will tighten the limit in (1) even further.

The surface wave resonances on a finite MTS antenna can be qualitatively determined by:

6 $$ \beta l = \pi $$

where *β* represents the propagation constant of the surface wave resonances, and *l* is the total length of the metasurface structure given by

7 $$ l = NP $$

where *N* represents the number of cells, and *P* is the periodicity of the metasurface. By substituting (7) to (6), we get

8 $$ \beta = \pi /NP $$

The propagating constant of the surface waves decaying away from the metasurface is related to the decay constant (*α*) and the frequency (*ω*) by^36^

9 $$ \beta = \sqrt {\eta^{2} \omega^{2} + \alpha^{2} } $$

The propagation constant for the transverse magnetic (TM) and transverse electric (TE) waves are given by

10 $$ \beta_{TM} = \frac{\omega }{c}\sqrt {1 - \frac{{Z^{2} }}{{\eta^{2} }}} $$

11 $$ \beta_{TE} = \frac{\omega }{c}\sqrt {1 - \frac{{\eta^{2} }}{{Z^{2} }}} $$

where *c* is the speed of light in a vacuum, *η* is the intrinsic impedance, and *Z* is the surface impedance of the MTS structure.

### Simulation results and experimental validation

**Figure 6:**
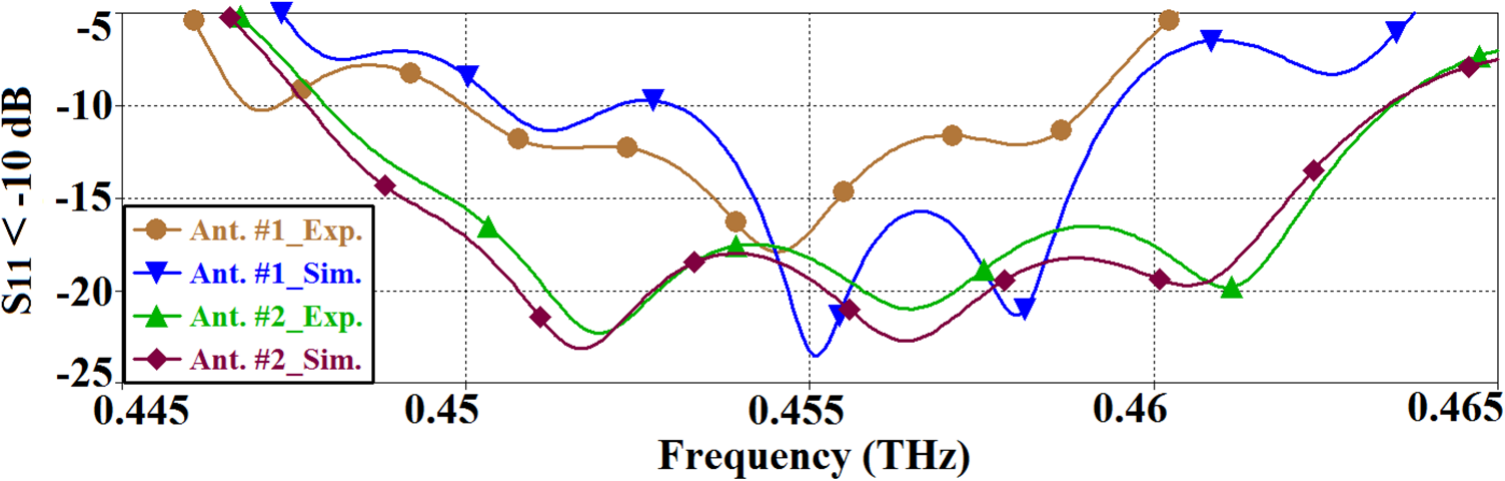
Simulated and measured reflection-coefficient response of the SIW-inspired AoC (Ant. #1) and the SIW-MTS inspired version (Ant. #2).

Figure 6 shows the simulated and measured reflection response of both the SIW inspired antenna (Ant. #1) and the SIW-MTS inspired version (Ant. #2). It is evident that by combing MTS with SIW technologies, the antenna’s operating bandwidth and the impedance matching performance are enhanced. The measured bandwidth of Ant. #1 is 9.2 GHz, however for Ant. #2 it is 15.4 GHz for |*S*_11_|≤ –10 dB. This constitutes a significant improvement of 67.4%. Moreover, the average measured impedance matching of Ant. #1 is 12.5 dB, and for Ant. #2 it is 18 dB over the bandwidth defined by |*S*_11_|≤ –10 dB, which constitutes and improvement of 5.5 dB. Good correlation between the simulated and measured results can be observed.

**Figure 7:**
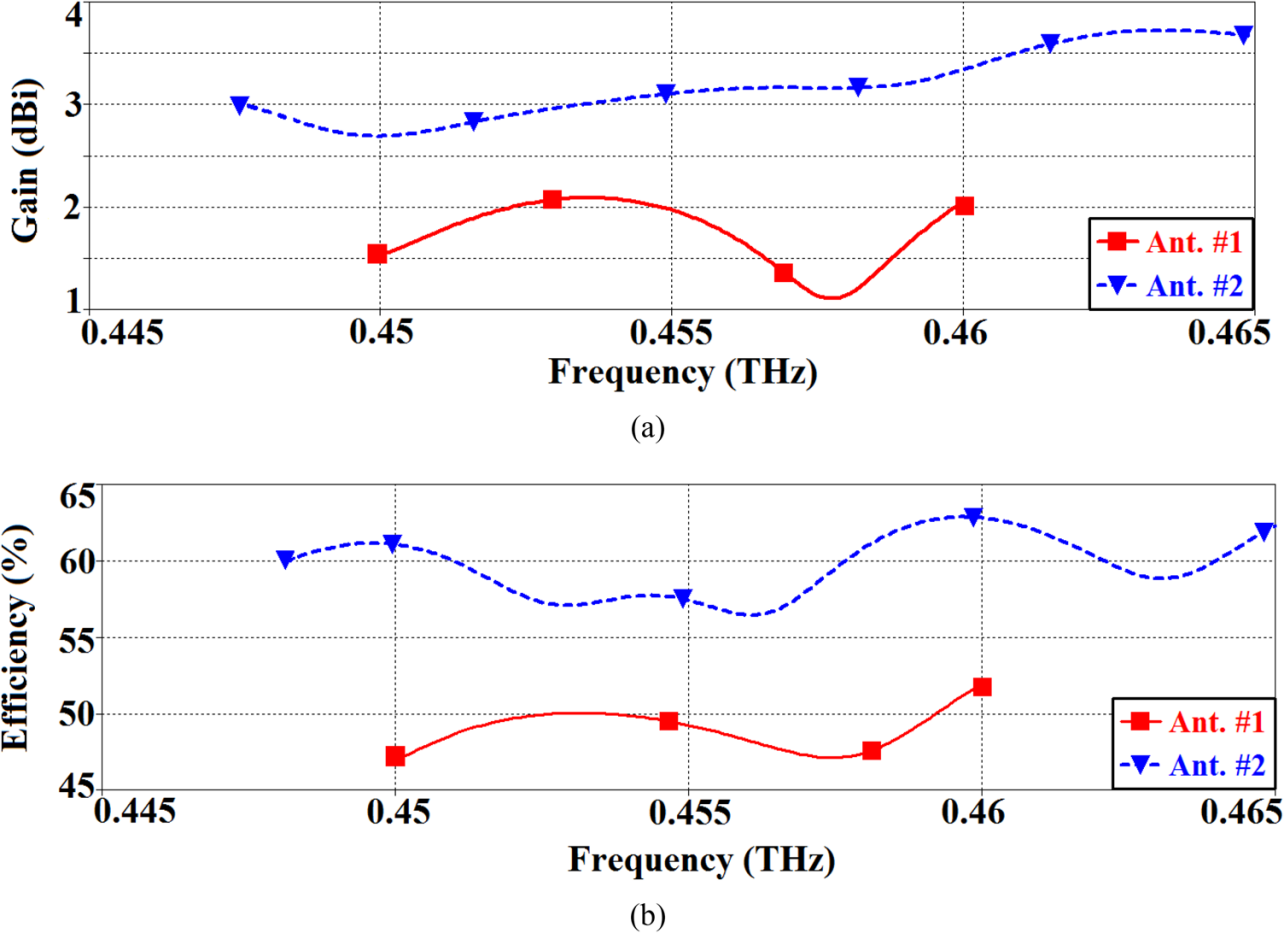
Antenna gain and radiation efficiency of Ants. #1 and #2 over their operating frequency spans.

The effectiveness of SIW-MTS approach on the radiation properties has been shown Fig. 7. It is evident from the measured results that by combining the two technologies the gain and radiation efficiency have been significantly boosted. The average gain and efficiency of Ant. #1 are 1.6 dBi and 48%, respectively; however, with application of both SIW and MTS technologies, the gain and efficiency on average improve to 3.2 dBi and 60%, respectively. The improvement in gain is 1.6 dBi, and the improvement in efficiency is 12%. These results demonstrate the benefit of amalgamating the two technologies in the design of on-chip antennas fabricated on GaAs. Moreover, the application of MTS does not complicate the antenna design and the antenna dimensions remain unchanged.

In the next section we investigate the application of MTS to the feedline, and its effects on the antenna performance.

## Impedance matched feedline of the SIW-MTS inspired AoC

**Figure 8:**
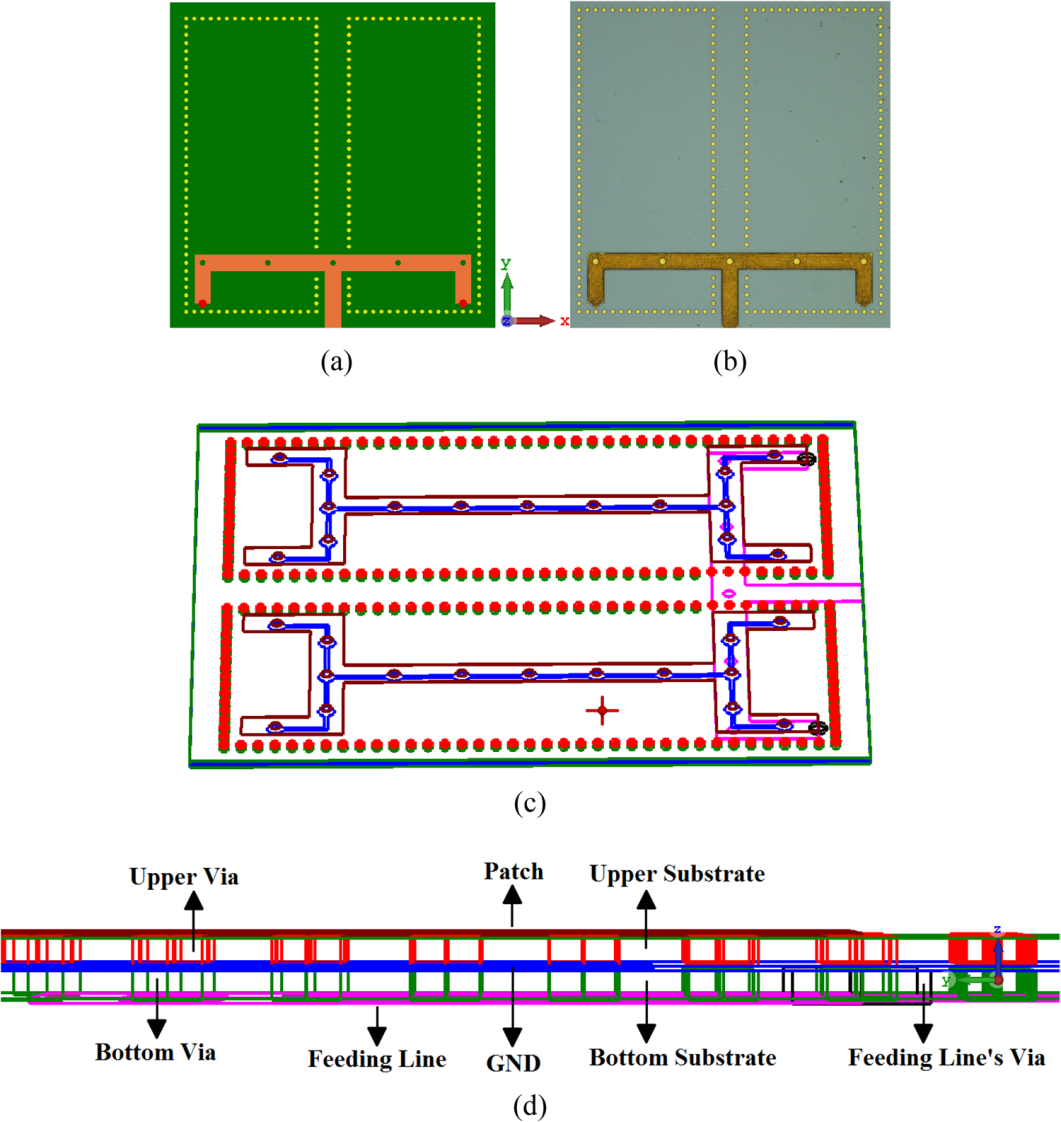
(**a**) Layout of the SIW-MTS inspired AoC with MTS short-circuited feedline, (**b**) fabricated GaAs prototype, (**c**) illustration showing positions of the metallic post wall around the wrench-shaped radiators and the ground-plane slots, and (**d**) cross-sectional view of the antenna’s five stacked layers.

The simplicity and the ease of implementing MTS in the antenna structure prompted us to ask the question how it will affect the antenna performance when applied to the T-shaped feedline on the underside of the bottom GaAs layer. It was found in^37^ that the impedance bandwidth of the antenna highly depends on the vertical offset position and the length of the horizontal strip in the T-shaped feedline. These parameters have been optimized in “SIW-inspired THZ antenna-on-chip” Section. Circular slots of identical diameters as those created on the radiating elements were implemented on the feedline. Also, the two ends of the T-shaped feedline are short-circuited to the ground plane using metallic vias, as shown in Fig. 8. The short circuit forces the standing wave to nullify at the two ends of the T-shaped feedline and thus forcing the EM-field to maximize in the middle section of the feedline arms. The position of the T-shaped feedline is aligned with one of the wrench-shaped radiator element’s ends to optimize EM coupling through the middle ground-plane layer slots. The dimensions of the slots are given in Table 2 where all other structural dimensions remain unaltered and are listed in Table 1.

**Table 2:** Dimensions of the slots (in microns & guide-wavelength).

Radius of circular slots on the radiating elements	3.5 μm	0.0191 *λ*_*g*_
Radius of feedline circular slots	3.5 μm	0.0191 *λ*_*g*_
Gap between the slots on the radiating elements	45 μm	0.2449 *λ*_*g*_
Gap between the slots on the feedline	70 μm	0.3810 *λ*_*g*_

**Figure 9:**
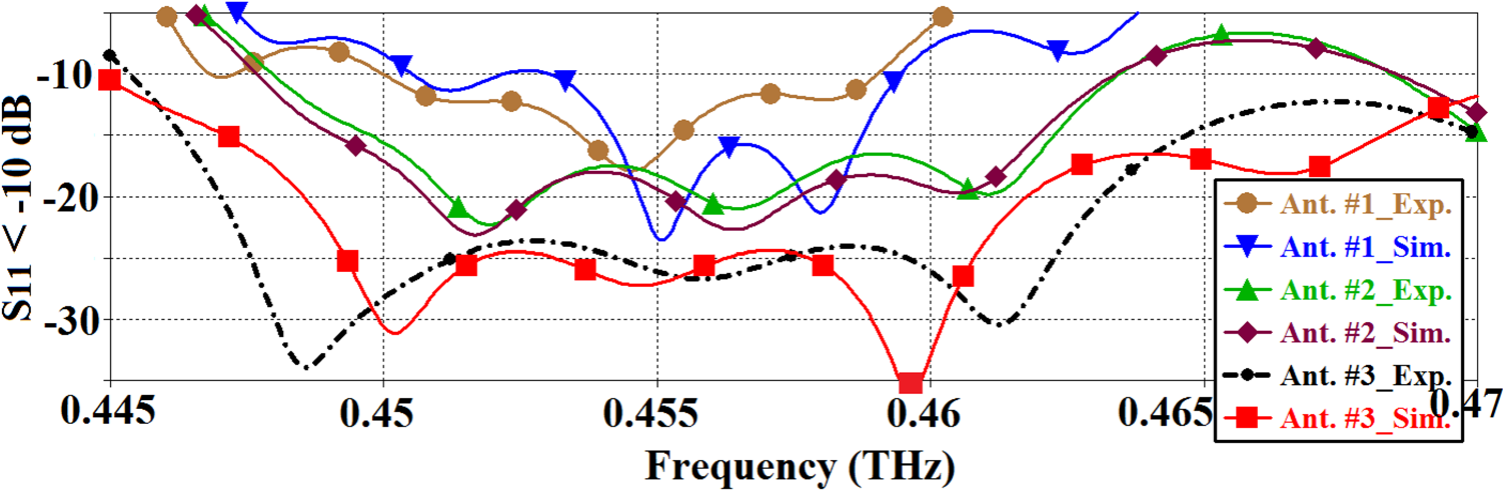
Comparison of the reflection-coefficient response of Ant. #1 (SIW), Ant. #2 (SIW-MTS), and Ant. #3 (SIW-MTS with feedline MTS and short-circuited feedline ends).

Figure 9 shows the performance improvements of the antenna with short-circuited feedline ends and MTS slots, referred to as Ant. #3, as compared to Antennas #1 and #2. It can be observed that the impedance bandwidth of Ant. #3 is substantially larger than that of Ant. #1 and #2. The measured impedance bandwidth of Ant. #3 is 25 GHz (0.445–0.470THz), Ant. #2 is 15 GHz (0.449–0.464) and Ant. #1 is 9 GHz (0.448–0.457). The impedance bandwidth of Ant. #3 is 67% greater than Ant. #2, and 178% greater than Ant. #1. The average measured values of impedance matching of Ant. #3, #2 and #1 are 21 dB, 15 dB and 13 dB. There is good agreement between the simulated and measured results. These results can be found in Table 3.

**Table 3:** Bandwidth & impedance match comparison of the three antennas.

	Improvement in impedance bandwidth (%)	Improvement in the average impedance matching (dB)
Ant. #2 vs. #1	66.7	2
Ant. #3 vs. #2	66.7	6
Ant. #3 vs. #1	177.8	8

**Figure 10:**
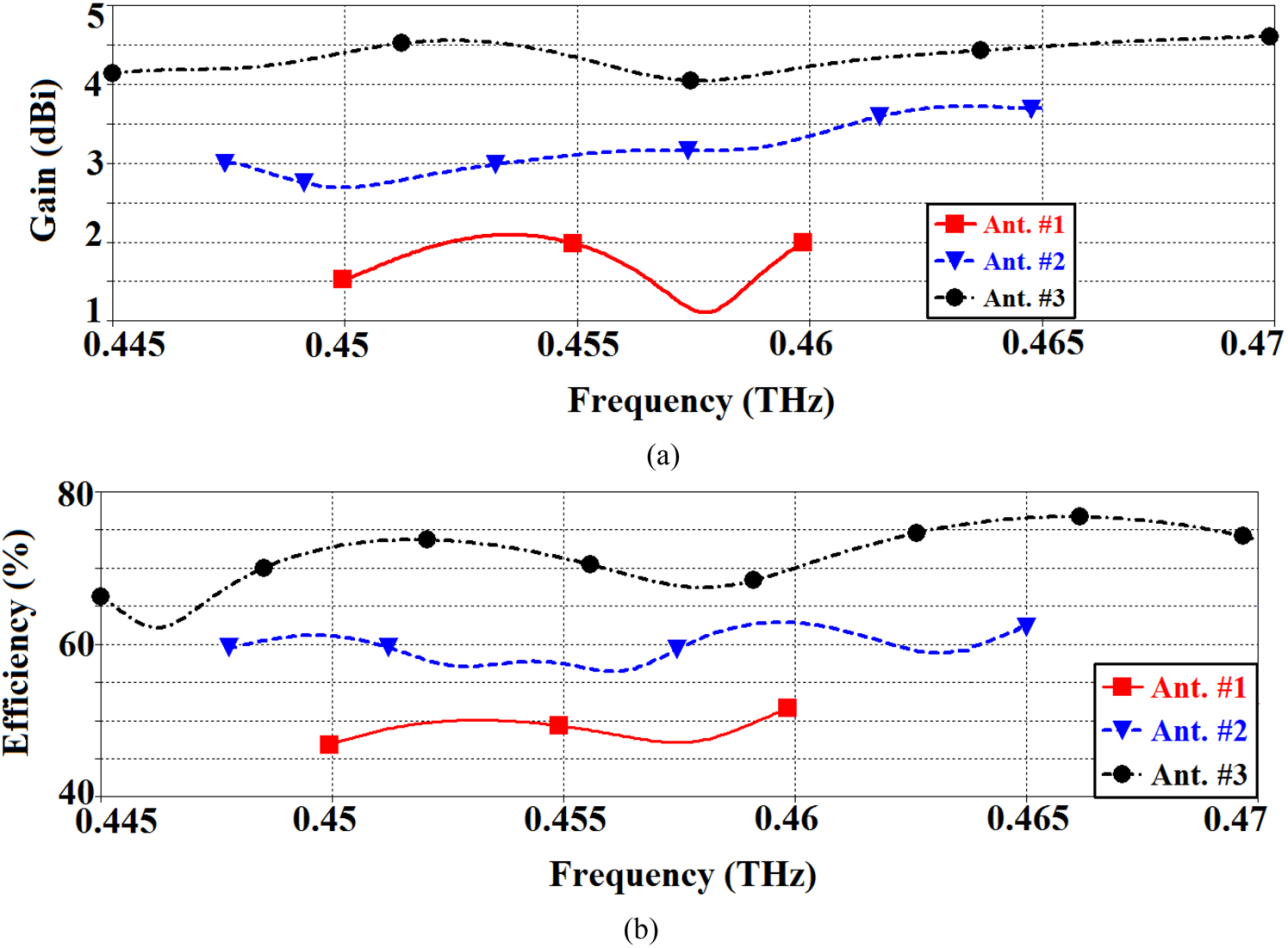
Comparison of the antenna gain and radiation efficiency response of Ant. #1 (SIW), Ant. #2 (SIW-MTS), and Ant. #3 (SIW-MTS with feedline MTS and short-circuited feedline ends).

**Figure 11:**
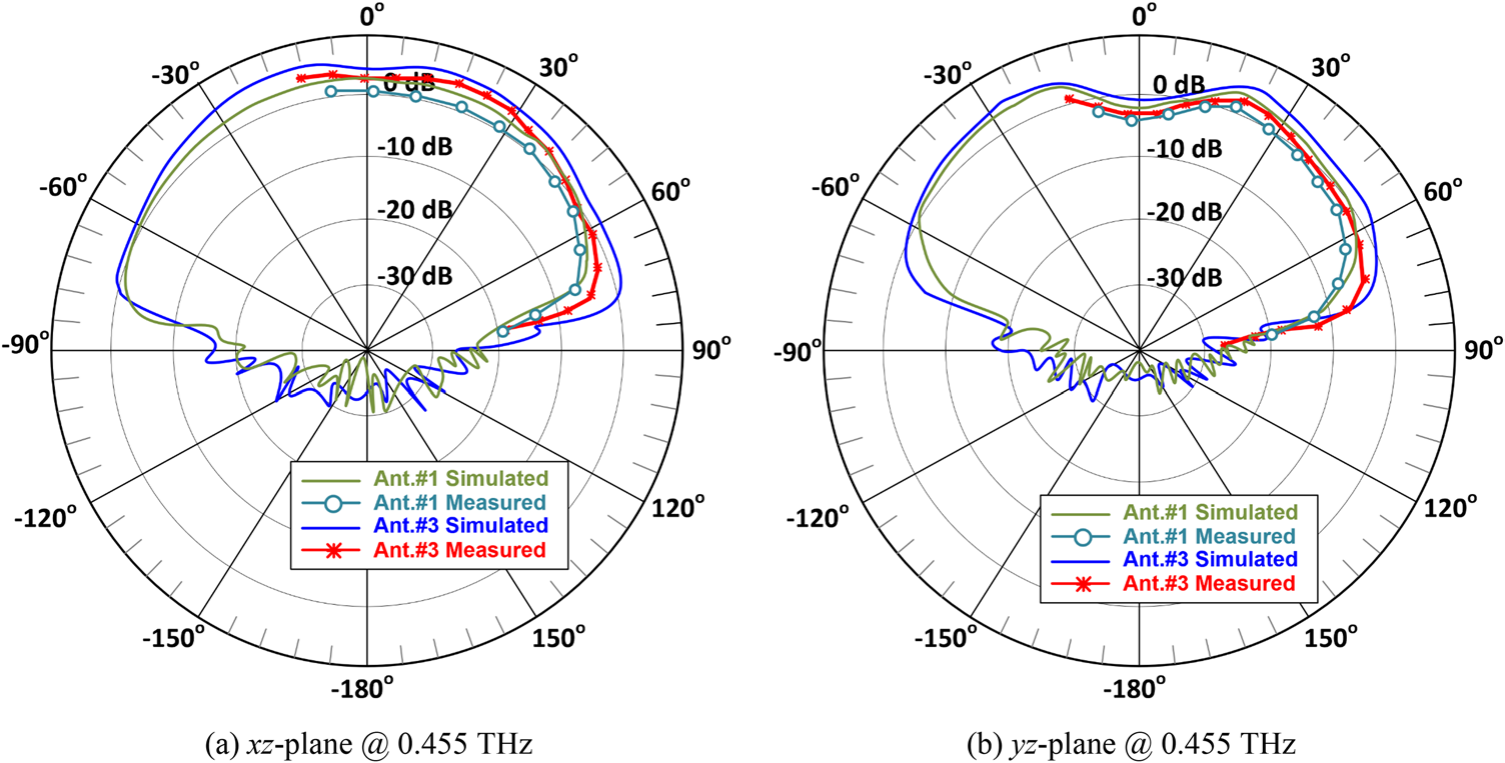
Simulated and measured radiation patterns of the proposed AoC in the orthogonal planes at 0.455 GHz.

The gain and radiation efficiency of the three antennas are shown in Fig. 10. Ant. #3 has the average gain of 4.8 dBi over the frequency range 0.445–0.47 THz. Ant. #2 is observed to have an average gain of 3.4 dBi, and Ant. #1 of 1.6 dBi. The average radiation efficiency of Ant. #3 is 73%, Ant. #2 is 60% and Ant. #1 is 50%. These results are summarized in Table 4. The radiation pattern of the AoC was measured using the setup shown in Fig. 3. The simulated and measured radiation patterns of Ant. #1 and Ant. #3 at the antenna’s central operating frequency of 0.455 GHz are shown in Fig. 11. As the radiation from the AoC is blocked by the probe in certain directions and its supporting arm it was not possible to measure the complete radiation pattern. There is good correlation between the measured and simulated results.

**Table 4:** Gain & radiation efficiency comparison of the three antennas.

Ant	Improvement in gain (dB)	Improvement in efficiency (%)
Ant. #2 vs. #1	1.8	10
Ant. #3 vs. #2	1.4	13
Ant. #3 vs. #1	3.2	23

The next section provides a comparison of the proposed antenna-on-chip with state-of-the-art structures reported in the literature, as well as discussion of its practical applications in the THz domain.

## State-or-the-art comparison

The performance parameters of the proposed antenna-on-chip are compared with the recent literature in Table 5. The proposed SIW-MTS-inspired GaAs antenna operates at much higher THz frequency range (0.445–0.470 THz) compared to other antennas referenced in the table, except for^38^, which is fabricated on Silicon wafer. Compared to^38^, the proposed antenna (Ant. #3) exhibits marginally higher gain but significantly higher radiation efficiency of 73%. The dimensions of the proposed antenna are comparable to the structure of^38^. The comparison shows the effectiveness of applying combined SIW and MTS technologies in the realization of on-chip antennas.

**Table 5:** Proposed AoC compared with state-of-the-art designs.

Ref	Antenna type	BW (GHz)	Gain (dBi)	Eff. (%)	Size (mm^3^)	Substrate
^39^	Bowtie-slot	90–105	≤ − 1.78	–	0.71 × 0.3 × 0.65	Si
^40^	Differential-fed circularly polarized	50–70	≤ − 3.2	–	1.5 × 1.5 × 0.3	Si
^41^	Ring-shaped monopole	50–70	≤ 0.02	≤ 35	–	Si
^42^	Circular open loop	57–67	≤ − 4.4	–	1.8 × 1.8 × 0.3	Si
^43^	AMC embedded squared slot	15–66	≤ 2	–	1.44 × 1.1	Si
^44^	Monopole	45–70	≤ 4.96	–	1.9 × 1.9 × 0.25	Si
^45^	Loop	65–69	≤ 8	≤ 96	0.7 × 1.25	Si
^46^	Dipole	95–102	≤ 4.8	–	–	Si
^47^	Tab monopole	45–75	≤ 0.1	≤ 42	1.5 × 1	Si
^48^	Transmitter and receiver modules	218–246	~ 8.5	–	2.74 × 0.7 × 0.15	Si
^38^	Metamaterials and dielectric resonators	450–475	≤ 4.5	≤ 45	0.4 × 0.4 × 0.135	Si
^49^	Monopole	290–320	≤ 1.72	–	0.39 × 0.3 × 0.78	InP
This work	SIW-MTS inspired	445–470	4.6	74	0.4 × 0.4 × 0.008	GaAs

## Conclusion

This paper demonstrates, for the first time, the design and realization of an on-chip antenna by utilizing substrate integrated waveguide (SIW) and metasurface (MTS) technologies. The antenna comprises a stack of five interleaved GaAs and metal layers. Dual wrench-shaped radiating metal elements are implemented on the top side of the upper GaAs layer, whose bottom side is a common metal ground plane. The two radiating elements are contained inside a wall of metal posts that protrude through the GaAs layers. The antenna is excited from underneath using a T-shaped feedline implemented on the underside surface of the bottom GaAs layer. The radiating elements, and the T-shaped feedline are transformed into a metasurface by embedding on them a periodic array of circular slots of sub-wavelength diameter and periodicity. Electromagnetic energy from the feedline is coupled to the radiating elements through the slots in the middle ground-plane layer. It is shown that the proposed antenna operates over a wide frequency range from 445 to 470 GHz with the average impedance matching, gain, and radiation efficiency of 21 dB, 4.6 dBi, and 74%, respectively. To the best authors’ knowledge, these performance figures make the proposed antenna competitive over state-of-the-art designs available to date.

## Data Availability

All data generated or analyzed during this study are included in this article.
